# Supplementary material for: Evaluation of Alphafold modeling for elucidation of nanobody-peptide epitope interactions
Source: bioRxiv. 2025 Mar 18:2025.03.14.643405. Preprint. [Version 2] doi: 10.1101/2025.03.14.643405 (PMC12478407; doi:10.1101/2025.03.14.643405)
Supplement: Supplement 1 [file media-1.pdf]

Supporting Information for –

Evaluation of Alphafold modeling for elucidation of nanobody-peptide epitope interactions

Supporting Methods:

Input protein sequences.

Nb$_{6E}$:
QVQLQESGGGLVQPGGSLRLSCAASGFVFENSAMAWYRQAPGKERELIAVIGTTFIKLAESVK GRFTISR DNAKSTVYLQ MNNLKPEDTA VYYCSKSGAYWGQGTQVTVSS

Nb$_{Alfa}$:
EVQLQESGGGLVQPGGSLRLSCTASGVTISALNAMAMGWYRQAPGERRVMVAAVSERGNA MYRESVQGRFTVTRDFTNKMVSLQMDNLKPEDTAVYYCHVLEDRVDSFHDYWGQGTQVTVS S

Nb$_{headlock}$:
QVQLVESGGGLVQPGGSLTLSCTASGFTLDHYDIGWFRQAPGKEREGVSCINNSDDDTYYAD SVKGRFTI FMNNAKDTVYLQMNSLKPEDTAIYYCAEARGCKRGRYEYDFWGQGTQVTV SS

Nb$_{PepTag}$:
QVQLQESGGGLVQPGGSLRLSCAASGNIVSIDAAGWFRQAPGKQREPVATILTGGATNYADS VKGRFTISRDNAKNTVYLQM NSLKPEDTAVYYCYAPMIYY GGRYSDYWGQGTQVTVSS

Nb$_{127}$:
EVQLVESGGGLVQAGESLRLSCAASGSTFDFKVMGWYRQPPGKQREGVAAIRLSGNMHYAE SVKGRFAISKANAKNTVYL QMNSLRPEDTAVYYCKVNIR GQDYWGQGTQVTVSS

Nb$_{Moon}$:
EVQLVESGGGLVQPGGSLRLSCAASGSISSVDVMSWYRQAPGKQRELVAFITDRGRTNYKVS VKGRFTISRDNSKNMVYLQMNSLKPEDTADYLCRAESRTSWSSPSPLDVWGRGTQVTVSS

6E tag: QADQEAKELARQIS

Alfa tag: SRLEEELRRRLTE

BC2 (headlock) tag: PDRVRAVSHWSS

PepTag: AVERYLKDQQLLGIW

127 Tag: SFEDFWKGED

Moon tag: KNEQELLELDKWASL

UBC6E(1-184), 6E tag underlined:
METRYNLKSPAVKRLMKEAAELKDPTDHYHAQPLEDNLFEWHFTVRGPPDSDFDGGVYHGRI VLPPEYPM
KPPSIILLTANGRFEVGKKICLSISGHHPETWQPSWSIRTALLAIIGFMPTKGEGAIGSLDYTPEE RRAL
AKKSQDFCCEGCGSAMKDVLLPLKSGSDSSQADQEAKELARQIS

UBC6E(1-184)-127 tag, tag underlined:

METRYNLKSPAVKRLMKEAAELKDPTDHYHAQPLEDNLFEWHFTVRGPPDSDFDGGVYHGRI
VLPPEYPM
KPPSIILLTANGRFEVGKKICLSISGHHPETWQPSWSIRTALLAIIGFMPTKGEGAIGSLDYTPEE
RRAL
AKKSQDFCCEGCGSAMKDVLLPLKSGSDSSSFEDFWKGED

CXCR2(1-360), 127 tag underlined:
MEDFNMESDSFEDFWKGEDLSNYSYSSTLPPFLLDAAPCEPESLEINKYFVVIIYALVFLLSLLG
NSLVM
LVILYSRVGRSVTDVYLLNLALADLLFALTLPIWAASKVNGWIFGTFLCKVVSLLKEVNFYSGILLL
ACI
SVDRYLAIVHATRTLTQKRYLVKFICLSIWGLSLLLALPVLLFRRTVYSSNVSPACYEDMGNNTA
NWRML
LRILPQSFGFIVPLLIMLFCYGFTLRTLFKAHMGQKHRAMRVIFAVVLIFLLCWLPYNLVLLADTL
MRTQ
VIQETCERRNHIDRALDATEILGILHSCLNPLIYAFIGQKFRHGLLKILAIHGLISKDSLPKDSRPSF
VG
SSSGHTSTTL

CXCR2(1-360)-6E tag, 6E tag underlined:
MEDFNMESDQADQEAKELARQISLSNYSYSSTLPPFLLDAAPCEPESLEINKYFVVIIYALVFLLS
LLGNSLVM
LVILYSRVGRSVTDVYLLNLALADLLFALTLPIWAASKVNGWIFGTFLCKVVSLLKEVNFYSGILLL
ACI
SVDRYLAIVHATRTLTQKRYLVKFICLSIWGLSLLLALPVLLFRRTVYSSNVSPACYEDMGNNTA
NWRML
LRILPQSFGFIVPLLIMLFCYGFTLRTLFKAHMGQKHRAMRVIFAVVLIFLLCWLPYNLVLLADTL
MRTQ
VIQETCERRNHIDRALDATEILGILHSCLNPLIYAFIGQKFRHGLLKILAIHGLISKDSLPKDSRPSF
VG
SSSGHTSTTL

Supporting Tables.

Supporting Table 1. Summary of nanobodies and epitopes used in modeling studies.

Nanobody name	Structure (PDB code)	Release date	Pubmed ID(s)	Tag name	Tag sequence	Modelling success summary
VHH05 /VHH6E/ Nb6E	None	-	35930411, 31518855	6E tag	QADQEAKELARQIS (ubc6e)	Predicts important residues
Alfa	6I2G	9/25/2019	31562305	Alfa	SRLEEELRRRLTE	Very good match
Headlock	5IVN	4/6/2016	26791954, 29500346	BC2	PDRVRAVSHWSS (beta catenin)	Correct general site, some divergence in details
Peptag Nb, 2E7	5HM1 (no paper)	1/25/2017	32868807	Peptag	AVERYLKDQQLLGIW (from HIV GP41)	Only top model is highly accurate, others diverge
127d01	None (reported here)	-	35076390	CXCR2 ECD	SFEDFWKGED (human CXCR2)	Good match except for peptide C-terminus
Moon, 2H10	4B50 (no ligand), 7AEJ (full prot)	3/27/2013, 5/26/2021	31178119	Moon	KNEQELLELDKWASL (from HIV GP41)	Poor prediction

Supporting Table 2. Computational confidence parameters for AF2 models and comparison to experimental structures.

					Rank 1 comparison with experimental[1]		
**Complex**	**Rank (ColabFold)**	**pLDDT**	**pTM**	**ipTM**	**I-RMSD, Å**	**Peptide RMSD, Å**	**Model Accuracy**
NbAlfa	1	97.2	0.917	0.913	0.33	0.46	High
	2	97.2	0.917	0.912			
	3	97	0.915	0.911			
	4	97.2	0.917	0.909			
	5	96.7	0.914	0.91			
NbHeadlock	1	81.1	0.814	0.409	2.28	3.62	Acceptable
	2	80.7	0.81	0.361			
	3	80.2	0.81	0.358			
	4	80.3	0.806	0.269			
	5	80	0.809	0.254			
NbPepTag	1	90	0.872	0.841	1.82	1.5	Medium
	2	85	0.837	0.653			
	3	86.9	0.846	0.624			
	4	84.7	0.826	0.501			
	5	81.5	0.809	0.355			
NbMoon	1	83.9	0.828	0.458	2.68	4.45	Incorrect
	2	86.1	0.833	0.398			
	3	82.4	0.811	0.381			
	4	83.6	0.819	0.341			
	5	82.1	0.81	0.334			
Nb6E	1	97.4	0.918	0.914	No experimental structure available		
	2	97.3	0.92	0.913			
	3	97.3	0.919	0.91			
	4	97	0.917	0.909			
	5	96.7	0.915	0.904			
Nb127	1	92.2	0.89	0.849	0.47	1.17	Medium
	2	92.6	0.891	0.841			
	3	92.5	0.894	0.837			
	4	92.8	0.896	0.836			
	5	92.5	0.894	0.821			

[1]Model accuracy assessments for interface residue RMSD (I-RMSD), peptide RMSD, and CAPRI model accuracy level were computed based on comparison of model with corresponding X-ray structure using the DockQ program.[1]

Supporting Table 3. Complete data and statistics (diffraction data and refinement statistics) for X-ray analysis of Nb_{127}-127 tag complex crystal.

Data collection	Nb_{127}
ligand	127D01
Space group	P 31 2 1
Wavelength (Å)	1.54
Resolution (Å)	50-2.10
Highest resolution shell (Å)	2.14-2.10
Unit-cell parameters (Å)	a=46.876<br>b=46.876<br>c=91.184
α=β=**γ** (°)	90, 90, 120
Measurements	
Unique reflections	7180(369)
Redundancy	8.9(6.9)
Completeness (%)	99.3(98.4)[a]
Rmerg[b]	0.045(0.104)
<I/ $\sigma(I)$>	51.6(14.1)
**Refinement**	
No. of reflection (test)	7149(712)
Total atom (non-H)	1055
No. atoms	
Protein	900
ligand	89
Water	76
R	0.18
R_{free}	0.22
RMSD	
Bond lengths(Å)	0.003
bond angles (°)	0.60
Average B factor ($Å^2$)	
Subunit A	25.8
Subunit B	23.9
water	31.6
Ramachandran plot	
Favored (%)	100
Allowed (%)	0.00
Outliers (%)	0.00

[a]Figures in brackets apply to the highest-resolution shell.

[b]$R_{merg} = \Sigma_h\Sigma_i|I(h,i)-<I(h)>|/\Sigma_h\Sigma_i I(h,i)$, where $I(h,i)$ is the intensity of the *i*th observation of reflection *h*, and $< I(h)>$ is the average intensity of redundant measurements of reflection *h*.

Supporting Table 4: Assessment of side chain contributions to nanobody-tag interactions for Nb_{127}.

		Calc. ΔΔG (kJ/mol ± SD)			
**Residue**	**Amino Acid**	**Alphafold structures**			**Experimental (xtal strucuture)**
2	PHE	14.1	±	1.2	16.4
3	GLU	5.5	±	0.2	5.7
4	ASP	0.0	±	0.0	0
5	PHE	13.7	±	0.1	13
6	TRP	27.2	±	0.4	25.4
7	LYS	2.0	±	0.1	1.7
9	GLU	0.6	±	0.7	3.2
10	ASP	5.2	±	4.2	9.3

Supporting Table 5. Sequence and mass spec characterization of peptides used in study.

Peptide	Sequence	Calculated (M+H)	Observed (M+H)
127A1	H-AlaPheGluAspPheTrpLysGlyGluAsp-NH2	1242.54	1242.5
127A2	H-SerAlaGluAspPheTrpLysGlyGluAsp-NH2	1182.5	1182.5
127A3	H-SerPheAlaAspPheTrpLysGlyGluAsp-NH2	1200.5	1200.5
127A4	H-SerPheGluAlaPheTrpLysGlyGluAsp-NH2	1214.5	1214.6
127A5	H-SerPheGluAspAlaTrpLysGlyGluAsp-NH2	1182.5	1182.5
127A6	H-SerPheGluAspPheAlaLysGlyGluAsp-NH2	1143.5	1143.5
127A7	H-SerPheGluAspPheTrpAlaGlyGluAsp-NH2	1201.5	1201.5
127A8	H-SerPheGluAspPheTrpLysGluGluAsp-NH2	1330.6	1330.6
127A9	H-SerPheGluAspPheTrpLysGlyAlaAsp-NH2	1200.5	1200.5
127A10	H-SerPheGluAspPheTrpLysGlyGluAla-NH2	1214.5	1214.6
127C3	Ac-SerPheCysAspPheTrpArgGlyGluAsp-NH2	1301.5	1302.5
127X3	Ac-SerPheCys(xlink)AspPheTrpArgGlyGluAsp-NH2	1633.61	1634.6
FAM-127C3	FAM-SerPheCysAspPheTrpArgGlyGluAsp-NH2	1617.6	1619.6
FAM-127X3	FAM-SerPheCys(xlink)AspPheTrpArgGlyGluAsp-NH2	1949.7	976.3 (z=2)
6E-WT	H-GlnAlaAspGlnGluAlaLysGluLeuAlaArgGlnIleSer-NH2	794.0 (z=2)	793.9 (z=2)
6E-M9	H-GlnAlaAspGlnGluAlaLysGluMetAlaArgGlnIleSer-NH2	887.9 (z=2)	887.9 (z=2)
6E-F9	H-GlnAlaAspGlnGluAlaLysGluPheAlaArgGlnIleSer-NH2	810.9 (z=2)	810.9 (z=2)
6E-W4	H-GlnAlaAspTrpGluAlaLysGluLeuAlaArgGlnIleSer-NH2	908.4 (z=2)	908.5 (z=2)
6E-W7	H-GlnAlaAspGlnGluAlaTrpGluLeuAlaArgGlnIleSer-NH2	908.0 (z=2)	908.0 (z=2)
6E-W8	H-GlnAlaAspGlnGluAlaLysTrpLeuAlaArgGlnIleSer-NH2	907.5 (z=2)	907.5 (z=2)
G3-6E	H-GlyGlyGlyGlnAlaAspGlnGluAlaLysGluLeuAlaArgGlnIleSer-NH2	879.0 (z=2)	879.0 (z=2)

Supporting Table 6: Scores and accuracy assessments of Nb-tag complexes using AlphaFold2 run locally.

Nanobody name	pTM	ipTM	Model confidence	I-pLDDT	I-RMSD, Å[1]	Peptide RMSD, Å[1]	Model Accuracy[1]
VHH05/VHH6E/Nb6E[2]	0.92	0.91	0.91	97.63	-	-	-
Alfa	0.92	0.92	0.92	97.91	0.4	0.61	High
Headlock	0.85	0.49	0.56	75.46	1.68	2.17	Acceptable
Peptag Nb, 2E7	0.89	0.91	0.9	91.29	2.05	1.66	Medium
127d01	0.9	0.89	0.89	97.22	2.27	3.91	Acceptable
Moon, 2H10	0.84	0.39	0.48	72.44	8.79	14.75	Incorrect

[1]Model accuracy assessments for interface residue RMSD (I-RMSD), peptide RMSD, and CAPRI model accuracy level were computed based on comparison of model with corresponding X-ray structure using the DockQ program (1).

[2]No X-ray structure is available for this complex, so accuracy assessments were not possible to calculate (noted as “-“).

Supporting Figures.

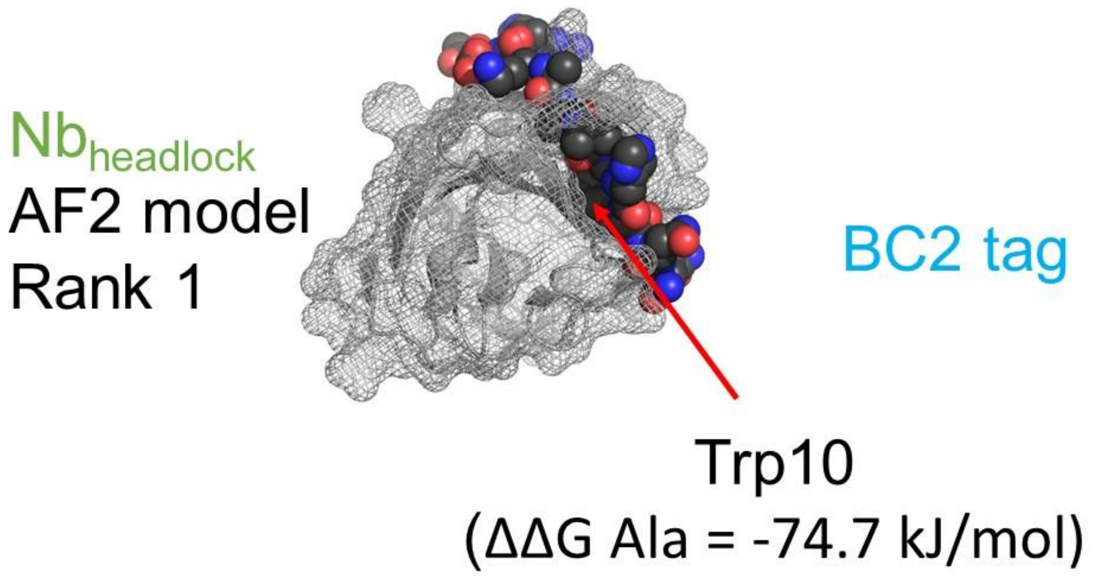

Supporting Figure 1. Illustration of steric clashes generated in AF2 models. Top ranking AF2 models for the Nb$_{headlock}$ and Nb$_{PepTag}$ complexes are shown with nanobody depicted in mesh and the binding peptide tag shown as spheres. Residues showing a negative ΔΔG Ala value in computational Ala scan and their steric clashes with the Nb surface are highlighted with arrows.

A) Nb-6E

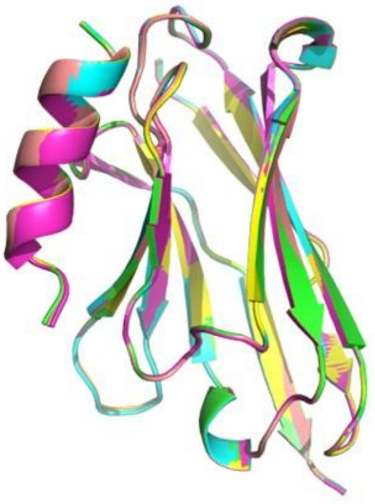

B) Computational analysis Alphafold

Residue	Amino Acid	ΔΔG (kJ/mol)		SD
1	GLN	2.5	±	0.2
3	ASP	11.9	±	0.3
4	GLN	0.0	±	0.0
5	GLU	12.0	±	0.7
7	LYS	1.6	±	0.0
8	GLU	0.0	±	0.0
9	LEU	8.9	±	0.7
11	ARG	0.1	±	0.0
12	GLN	2.8	±	0.3
13	ILE	9.4	±	0.1

Experimental: Large, medium, or small role in binding (Ref Cabalteja *et al.* ACS Chemical Biology 2022)

Supporting Figure 2. Comparison of AF2 predicted structures to experimental observations. A) Overlay of five AF2 models generated for the Nb_{6E}-6E tag complex. B) Computational Ala scan analysis peptide tag residues contributing to binding of AF2 models. Each of the five AF2 models was analyzed via computational Ala scan. Values shown represent mean ± SD upon analysis of all models.

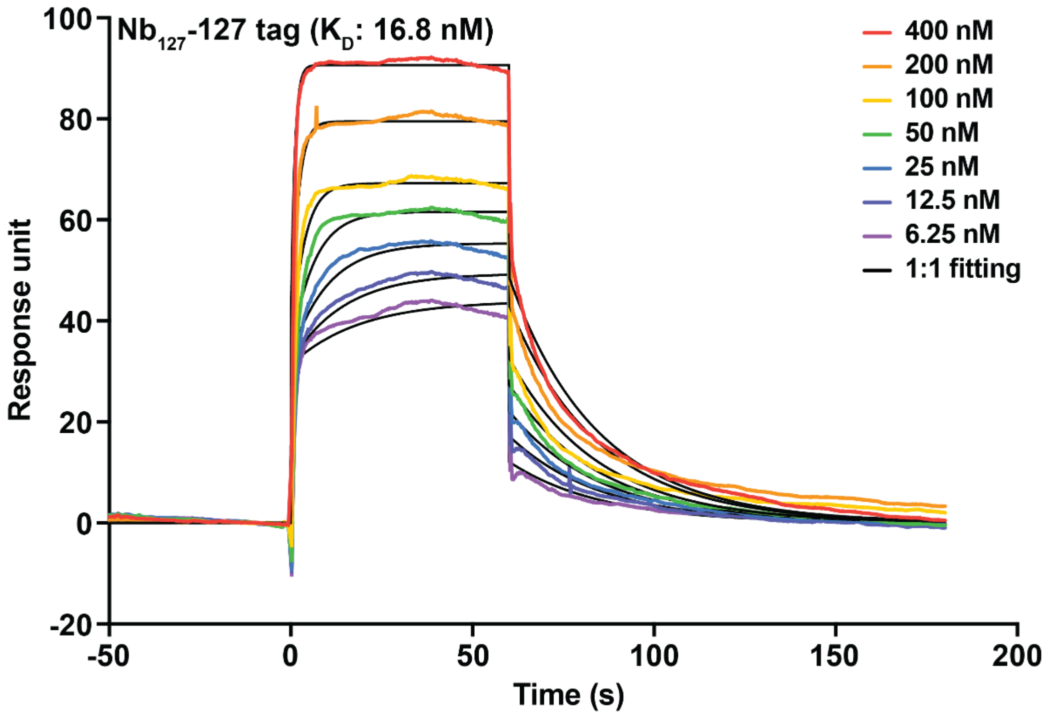

Supporting Figure 3. Characterization of Nb_{127}-127 tag affinity using surface plasmon resonance. Representative SPR sensorgram showing association and dissociation curves for interaction of 127 tag (400 – 6.25 nM) with Nb_{127}-biotin immobilized on a streptavidin (SA) chip (Cytiva, #29104992). Experimental details are described in **Methods**.

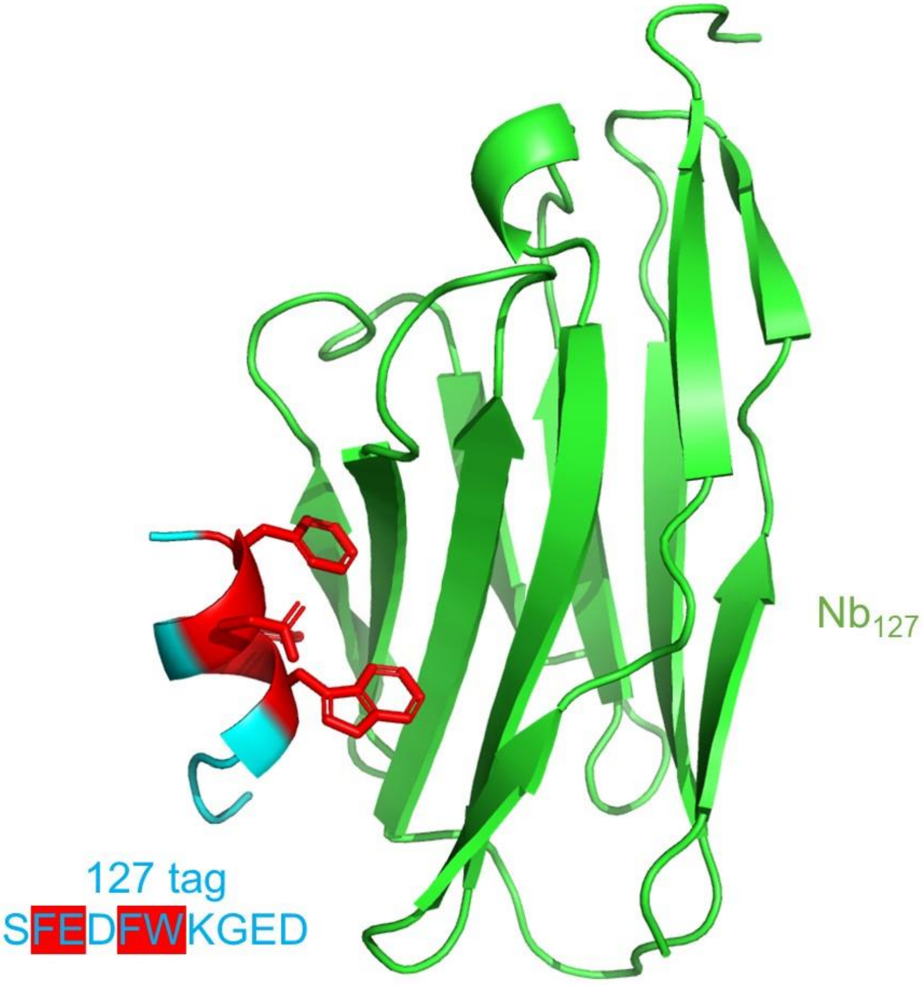

Supporting Figure 4. Illustration of residues important for binding withing the Nb127-tag experimental structure. Within the 127-tag peptide (cyan) residues shown to be important for binding in an ELISA are highlighted in red.

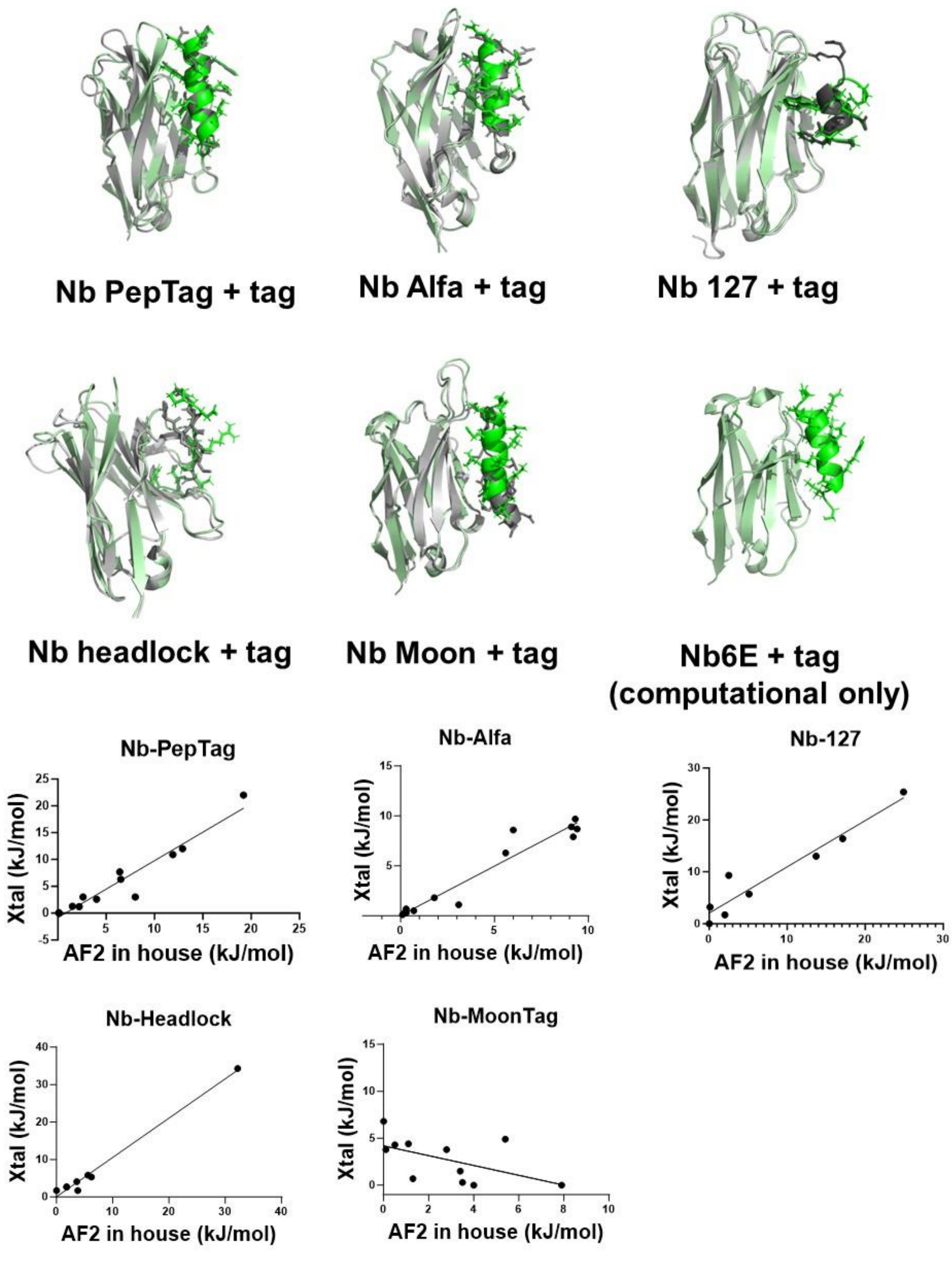

Supporting Figure 5. Overlay of experimental structures and top ranked models from application of *in house* AF2 implementation. (Top) Experimental models (Nb in light gray, tag in dark gray) were aligned with top ranked AF2 models (Nb in light green, tag in green) in Pymol. Tag peptides are shown with side chains illustrated with sticks. (Bottom) Quantitative assessment of peptide epitope side chain energetic contributions to binding. The energetic contribution of each side chain within experimental or the top ranked AF2 generated complex was quantified using BUDE Ala Scan. Each side chain was plotted as a single point in a scatter plot.

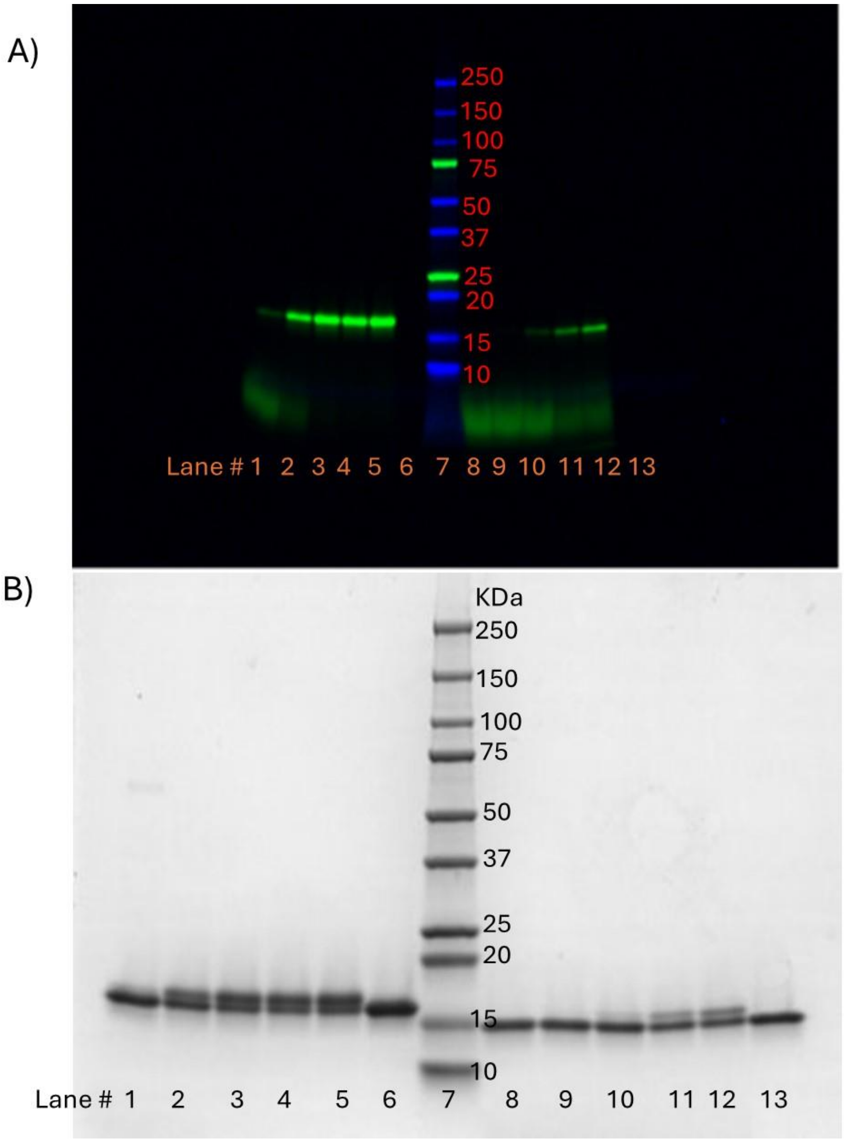

Lane# 1-6- Nb_{127D01} + FAM-127X3

#1 0 min
#2 5 min
#3 30 min
#4 120 min
#5 240 min
#6 Nb_{127D01}
#7 Ladder

Lane# 8-13- Nb_{6E} + FAM-127X3

#8 0 min
#9 5 min
#10 30 min
#11 120 min
#12 240 min
#13 Nb_{6E}

Supporting Figure 6: Comparison of the labeling of Nb_{127} and Nb_{6E} with FAM-127X3. Labeling reactions were performed with a mixture of indicated nanobodies (10 µM) and FAM-127X3 peptide (20 µM) in PBS at 25° C. The aliquots from the reaction mixture were collected and quenched at indicated time points using 4x SDS-PAGE sample buffer (Biorad # 1610747) containing 100 mM DTT. Reactions were then resolved using electrophoresis on a 4-20% acrylamide gradient gel (BioRad, #4561093). The gel was first scanned to detect the fluorescence (488 nm) and then stained with Coomassie blue for determination of total protein content. Representative gel images of a **A**) fluorescence scan (488 nm) and **B**) Coomassie staining for indicated reactions with conditions described above.

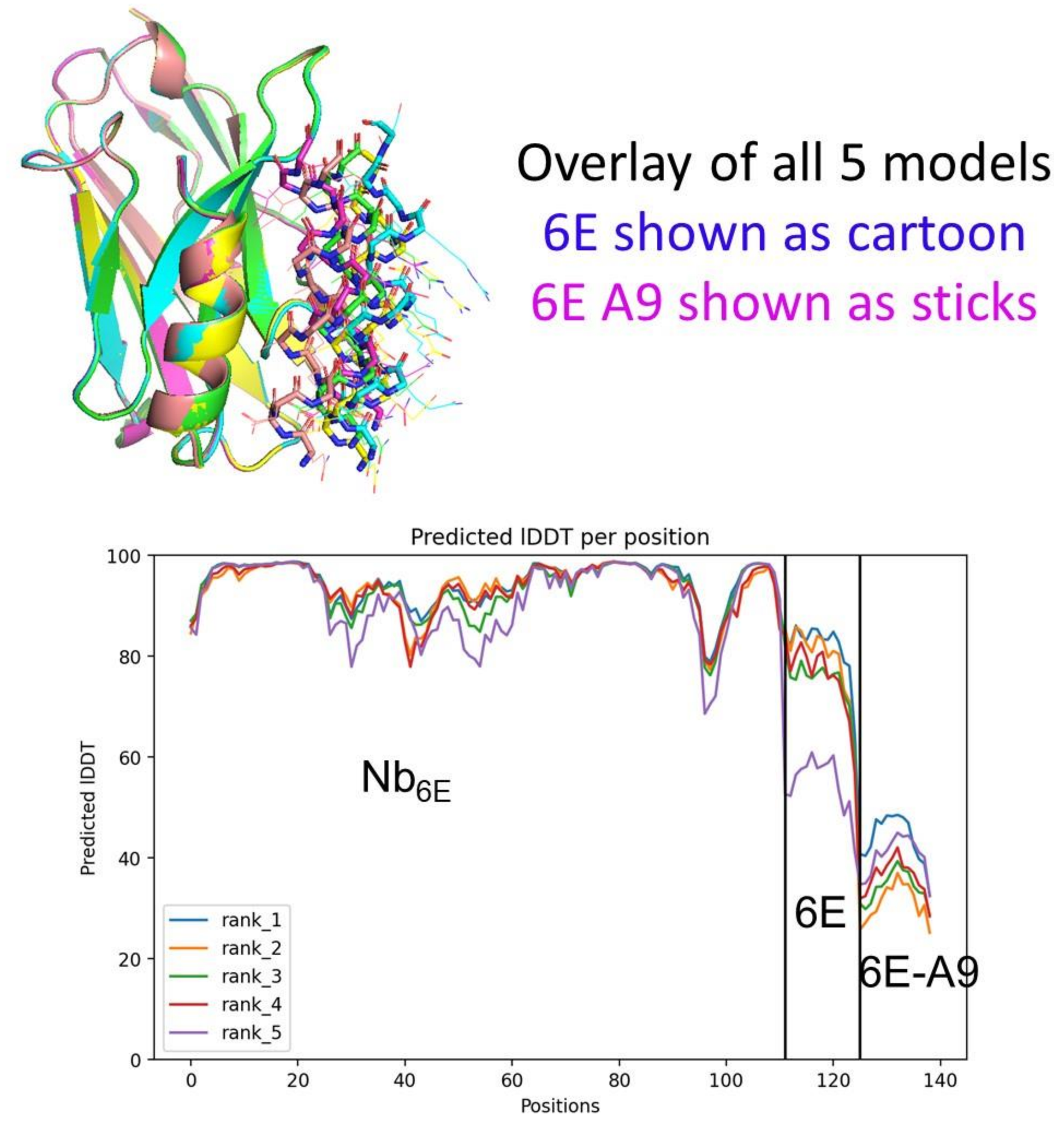

Supporting Figure 7. Comparison of peptide localization among AF2 models within peptide competition-based (6E vs 6E-A9). (Upper) An overlay of all 5 AF2 models (each its own color) generated for the tripartite complex consisting of Nb_{6E}, 6E, and 6E-A9 is shown. Nb_{6E} and 6E show a high degree of overlap among all models and are shown as cartoons. 6E-A9 shows weaker overlap and is shown as sticks. (Lower) pIDDT plot generated by Colabfold analyzing confidence in local structures across the predicted complex. Note the higher confidence levels shown for 6E relative to 6E-A9.

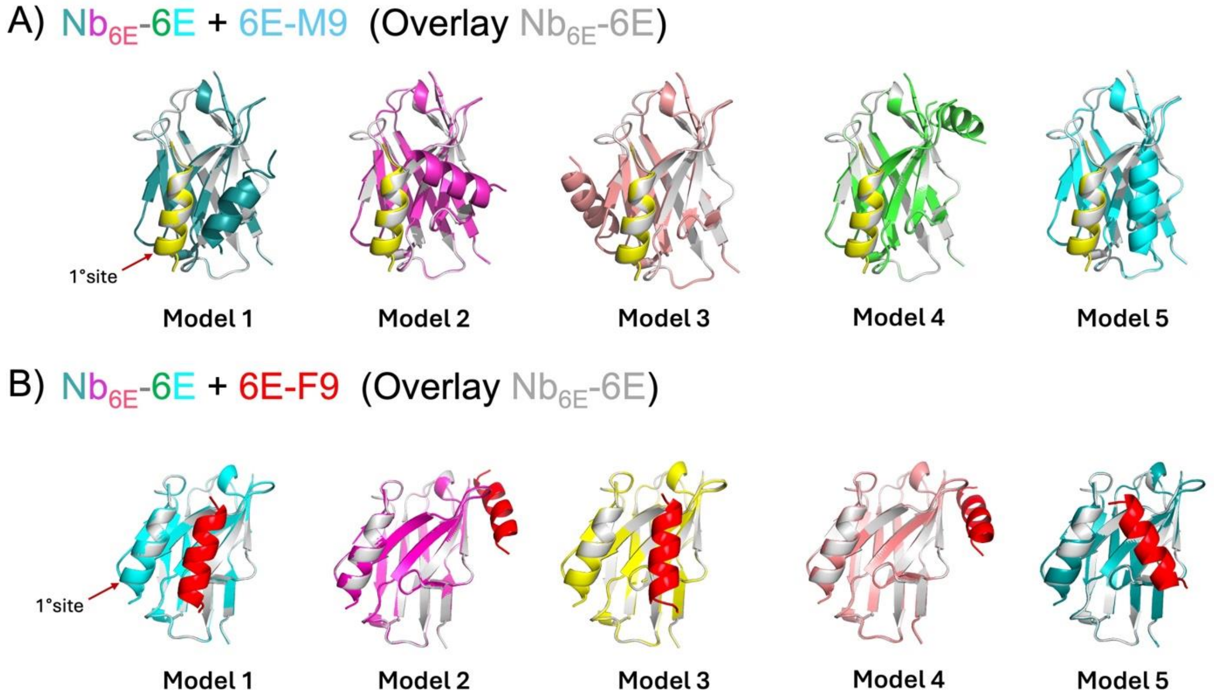

Supporting Figure 8. Evaluation of binding for 6E mutants via competition-based complex formation using AF2 models. Individual AF2 generated models were aligned and overlayed with a control model where 6E peptide occupies the primary (1°) binding site on the Nb_{6E} surface (in grey). **A)** 6E-M9 (yellow) outcompetes 6E (multicolor) for the 1° binding site on Nb_{6E} in all models. **B)** 6E-F9 (red) does not outcompete 6E (multicolor) for the 1° binding site on Nb6E.

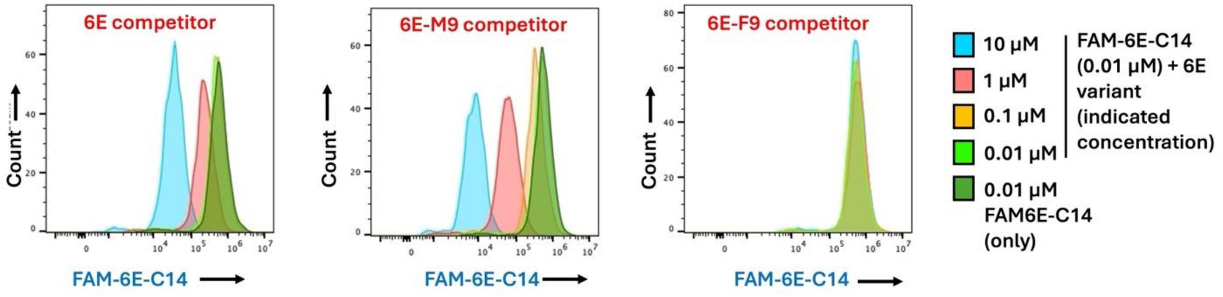

Supporting Figure 9. Competition binding assays to measure 6E peptide binding. Representative histograms from flow cytometry assays to measure 6E variant peptide binding to HEK293 cells stably expressing a cell surface protein fused with Nb_{6E} (A2AR-Nb_{6E}-ALFA). Cells were co-incubated with variable concentrations of variant 6E peptides (10 – 0.01 μM) and a fixed concentration (10 nM) of fluorescein-labelled 6E peptide (FAM-6E-C14), followed by washing, and detection with anti-fluorescein antibody conjugated with AF647 (see **Methods**). Histograms depict the intensity of AF647 signaling on live cells from a single representative experiment. These data were used to construct the dose-response shown in **Figure 5c** in the main text.

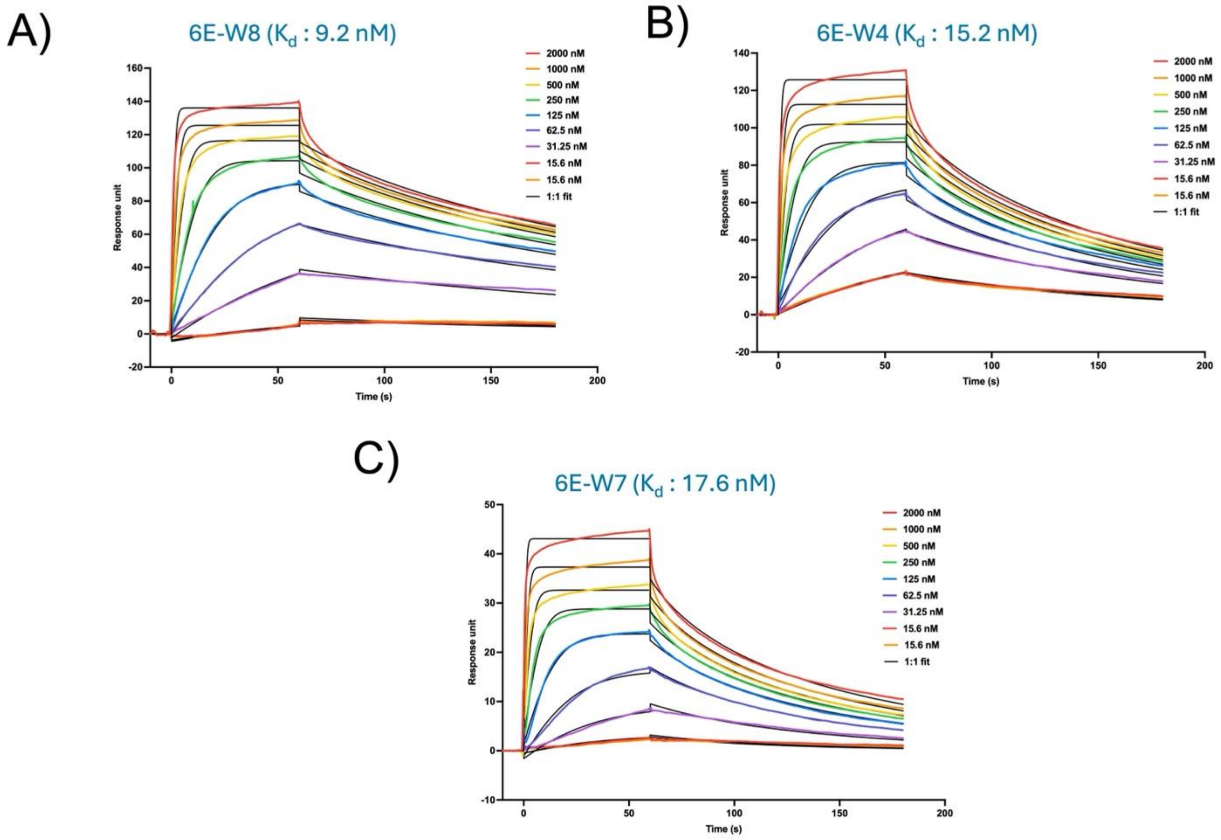

Supporting Figure 10. Quantitation of binding affinity for 6E variant peptides using SPR. Representative surface plasmon resonance sensorgrams of 6E variant peptides binding to immobilized Nb_{6E}-biotin on a streptavidin chip (Cytiva, #29104992). Colored lines correspond to binding sensorgrams at various peptide concentrations and black lines show the curve-fit generated by local fittings using 1:1 kinetics binding model, which was used to calculate K_d values. Experimental details are described in Methods. Ligand concentrations assessed are as follows: **A)** 6E-W8 (2 - 0.02 μM); **B)** 6E-W4 (2 - 0.02 μM); **C)** 6E-W7 (2 - 0.02 μM).

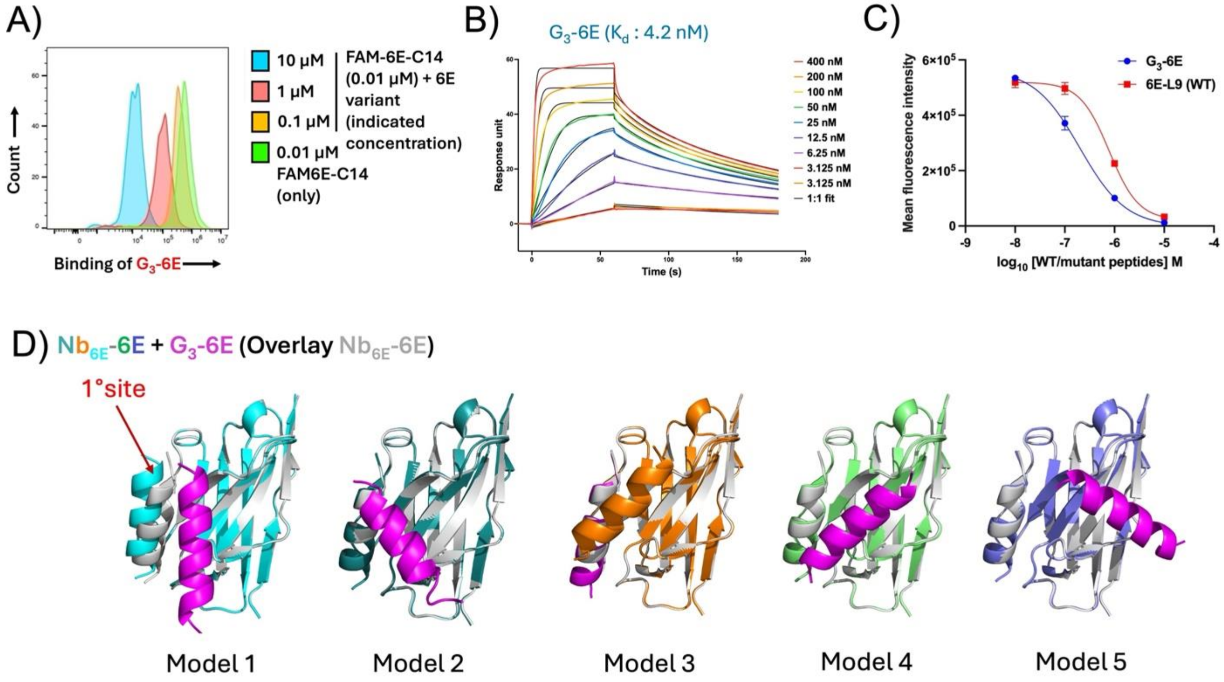

Supporting Figure 11. Assessment of G_3-6E peptide binding and AF modeling. A) Flow cytometry analysis of G_3-6E binding to cells stably expressing A2AR(Nb_{6E}) via competition with FAM-6E-C14. Cells were co-incubated with variable concentrations of G_3-6E (10–0.01 μM) and a fixed concentration (10 nM) of FAM-6E-C14, followed by washing, and detection with anti-fluorescein antibody conjugated with AF647 (see **Methods**). Histograms depict the intensity of AF647 signaling on live cells from a single representative experiment. **B)** Representative SPR sensorgram of G_3-6E (0.4 - 0.003 μM) binding to immobilized Nb_{6E}-biotin on a streptavidin coated chip (Cytiva, #29104992). Colored lines correspond to binding sensorgram at various ligand concentrations and black lines denote the curve-fit generated by local fittings using 1:1 kinetics binding model. Experimental details are described in **Methods**. **C)** Experimental flow cytometry competition binding assays to compare 6E-L9 (WT) and G_3-6E binding. HEK293 cells stably expressing a cell surface protein fused with Nb_{6E} (A2AR-Nb6E-ALFA) were co-incubated with variable concentrations of 6E-L9 (WT) and G_3-6E (10 – 0.01 µM) and a fixed concentration (10 nM) of fluorescein-labelled 6E peptide (FAM-6E-C14), followed by washing, and detection with anti-fluorescein antibody conjugated with Alexafluor647 (AF647) (see **Methods**). Binding was quantified as median AF647 fluorescence intensity. Note that the data for 6E-L9 (WT) is reproduced from main **Figure 5c**. **D**) Evaluation of binding for 6E mutants via competition-based complex formation using AF2 models. Individual AF2 generated models were aligned and overlayed with a control model where 6E peptide occupies the primary (1°) binding site on the Nb_{6E} surface (in grey). G_3-6E (magenta) fails to outcompete 6E (in multicolor) for the 1° binding site on Nb_{6E} in four out five models.

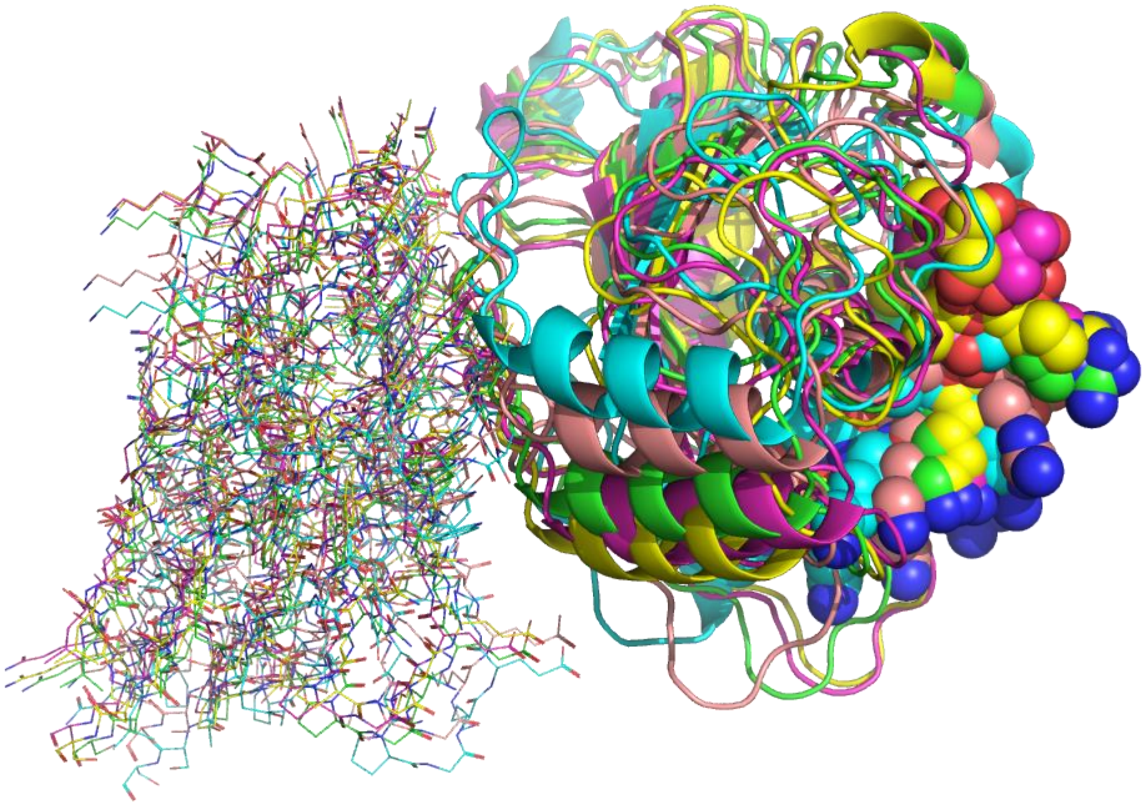

Supporting Figure 12. Visualization of AF2 models of Nb_{6E}-Ubc_{6E} complexes. Each of 5 AF2 models of is shown in a different color. For each complex Nb_{6E} is found at left shown as sticks, Ubc_{6E} in same color in the shown as cartoon, and the 6E tag in spheres at right. Models were generated by inputting sequences for Nb_{6E} and Ubc6E(1-184), which corresponds to the end of the 6E tag. Residues located C-terminally of the 6E tag are disordered were not included in modeling inputs.

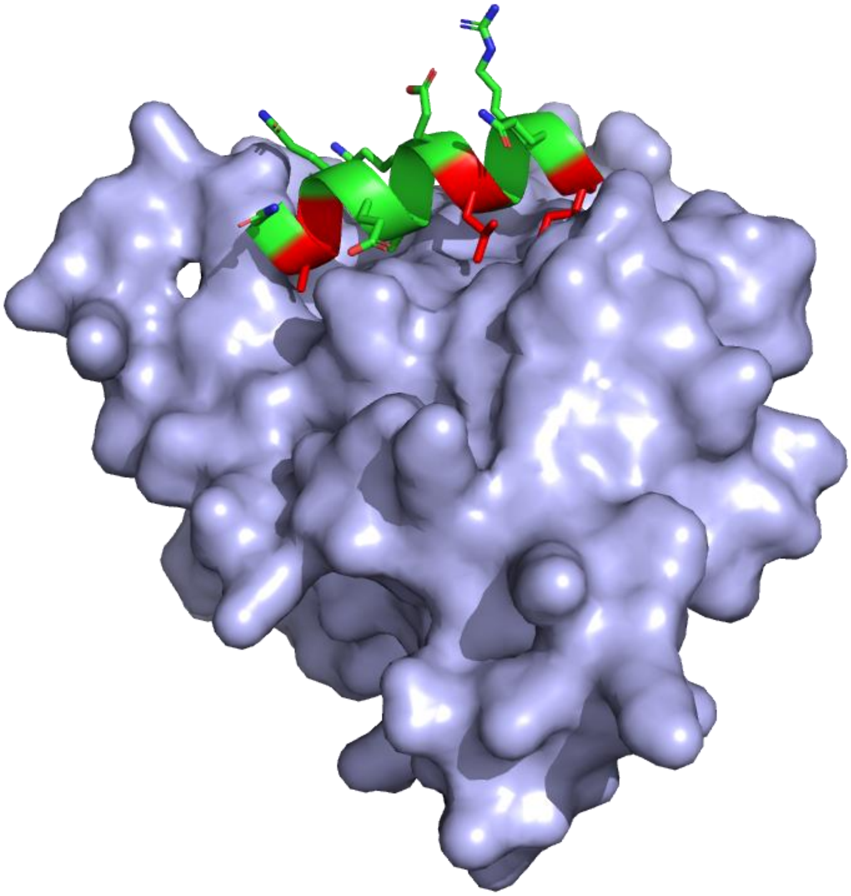

Supporting Figure 13. Illustration of burial of 6E sidechains in folded Ubc6E structure. An AF2 model of UBC6E structure is shown. UBC6E is shown as a blue surface except for the tag corresponding to the 6E tag portion, which is shown in green with residues important for binding to Nb6E highlighted in red.

References

1. Basu, S., andWallner, B. (2016) DockQ: A Quality Measure for Protein-Protein Docking Models PLoS One **11**, e0161879 10.1371/journal.pone.0161879
